# The use of equine chondrogenic‐induced mesenchymal stem cells as a treatment for osteoarthritis: A randomised, double‐blinded, placebo‐controlled proof‐of‐concept study

**DOI:** 10.1111/evj.13089

**Published:** 2019-04-13

**Authors:** S. Y. Broeckx, A. M. Martens, A. L. Bertone, L. Van Brantegem, L. Duchateau, L. Van Hecke, M. Dumoulin, M. Oosterlinck, K. Chiers, H. Hussein, F. Pille, J. H. Spaas

**Affiliations:** ^1^ Global Stem Cell Technology NV Anacura Group Evergem Belgium; ^2^ Department of Surgery and Anaesthesiology of Domestic Animals Faculty of Veterinary Medicine Ghent University Merelbeke Belgium; ^3^ Department of Veterinary Clinical Sciences Ohio State University Columbus USA; ^4^ Department of Pathology, Bacteriology and Poultry Diseases Faculty of Veterinary Medicine Ghent University Merelbeke Belgium; ^5^ Biometrics Research Group Faculty of Veterinary Medicine Ghent University Merelbeke Belgium

**Keywords:** horse, allogeneic, metacarpophalangeal joint, model, peripheral blood

## Abstract

**Background:**

There is a need to improve therapies for osteoarthritis in horses.

**Objectives:**

To assess the efficacy of equine allogeneic chondrogenic‐induced mesenchymal stem cells combined with equine allogeneic plasma as a novel therapy for osteoarthritis in horses.

**Study design:**

Randomised, double‐blinded, placebo‐controlled experiment.

**Methods:**

In 12 healthy horses, osteoarthritis was induced in the metacarpophalangeal joint using an osteochondral fragment‐groove model. Five weeks after surgery, horses were randomly assigned to either an intra‐articular injection with chondrogenic‐induced mesenchymal stem cells + equine allogeneic plasma (= intervention) or with 0.9% saline solution (= control). From surgery until the study end, horses underwent a weekly joint and lameness assessment. Synovial fluid was collected for cytology and biomarker analysis before surgery and at Weeks 5, 5 + 1d, 7, 9 and 11. At Week 11, horses were subjected to euthanasia, and the metacarpophalangeal joints were evaluated macroscopically and histologically.

**Results:**

No serious adverse events or suspected adverse drug reactions occurred during the study. A significant improvement in visual and objective lameness was seen with the intervention compared with the control. Synovial fluid displayed a significantly higher viscosity and a significantly lower glycosaminoglycan concentration in the intervention group. Other biomarkers or cytology parameters were not significantly different between the treatment groups. Significantly less wear lines and synovial hyperaemia were present in the intervention group. The amount of cartilage oligomeric matrix protein, collagen type II and glycosaminoglycans were significantly higher in the articular cartilage of the intervention group.

**Main limitations:**

This study assessed the short‐term effect of the intervention on a limited number of horses, using an osteoarthritis model. This study also included multiple statistical tests, increasing the risk of type 1 error.

**Conclusions:**

Equine allogeneic chondrogenic‐induced mesenchymal stem cells combined with equine allogeneic plasma may be a promising treatment for osteoarthritis in horses.

**The Summary is available in Spanish – see Supporting Information**

## Introduction

Osteoarthritis (OA) in horses often results in an early retirement from an athletic career or pleasure riding 1, 2, 3. Currently, treatment of OA is mainly focused on addressing the clinical signs 1, 3. The most commonly used treatments are corticosteroids, nonsteroidal anti‐inflammatory drugs, hyaluronan and polysulfated glycosaminoglycan 2. However, to date, none of these treatments halts the disease, let alone reverse it, so none of the current treatment modalities presents a durable solution for OA 4.

Regenerative medicine represents an interesting alternative for treating OA, since it has the potential to prevent further cartilage damage and even reverse the sustained damage 4, 5, 6, 7. Intra‐articular use of native mesenchymal stem cells (MSCs) has shown promising, though modest, results for enhancing cartilage repair 4, 5, 6, 7, 8. Moreover, the application of chondrogenic‐induced MSCs combined with plasma in horses with OA has been shown to have a clinical advantage compared with using plasma alone or native MSCs combined with plasma 7, 8. In those two studies, allogeneic MSCs (from another animal than the case, but from the same species) instead of autologous MSCs were used, as allogeneic MSCs allow ‘off‐the‐shelf’ therapy. However, randomised, double‐blinded, placebo‐controlled studies to objectively investigate efficacy of allogeneic MSCs are currently lacking.

Therefore, the goal of this study was to assess the efficacy of a single intra‐articular injection of equine allogeneic chondrogenic‐induced MSCs combined with equine allogeneic plasma in an experimental model of metacarpophalangeal (MCP) OA. We hypothesised that this combination would have a superior effect on OA improvement compared with saline (0.9%).

## Materials and methods

### Study design and animals

Twelve healthy warmblood horses, three geldings and nine mares (median age 8.5 years), were enrolled in this blinded, controlled, randomised and blocked study. The horses were free of visual lameness and presented no radiographic abnormalities of the MCP joints. The treatment was randomised using Randomizer_2 (2.1.0).xls. The horses were assigned to either the intervention group (n = 6) or to the control group (n = 6) per block of 2 horses (ratio 1:1).

### Intervention and control product

#### Intervention product

The intervention consisted of a proprietary formulation of equine allogeneic chondrogenic‐induced MSCs (ciMSCs) derived from peripheral blood (2 × 10^6^ cells in 1 mL of Dulbecco's modified Eagle medium low glucose with 10% of dimethyl sulfoxide)^a^ combined with equine allogeneic plasma (EAP) (1 mL plasma with 98 × 10^6^ platelets/mL) (Arti‐Cell^®^ Forte)^b^ . The ciMSCs and EAP were each stored in separate vials at −80°C until clinical application. Both donor horses (one for the ciMSCs and one for the EAP) were screened for 32 equine pathogens and collection of their blood was approved by the local ethics committee (approval number: EC_2012_001). These horses were not involved further in this study in any way. The IVP was produced according to Good Manufacturing Practice guidelines (BE/GMP/2015/082 and BE/GMP/2016/069) with a manufacturing authorisation (1868V) for veterinary medicinal cell‐based products.

#### Control product

The control product consisted of a sterile saline solution (0.9% NaCl)^c^ .

### OA joint model and exercise programme

Osteoarthritis was surgically induced in the right MCP joint of all horses at the study start (Week 0) as previously described 9. Briefly, the joint was arthroscopically assessed for the presence of pre‐existing cartilage lesions. These lesions were documented but were not an exclusion criterion. In the right MCP joint, a dorsomedial P1 osteochondral (OC) fragment was created that remained attached to the dorsal joint capsule and the fragment bed was debrided with an arthroburr to decrease apposition between fragment and fracture bed. Next, a horizontal groove in the cartilage on the dorsal aspect of the medial condyle of the third metacarpal bone was created using the arthroburr. Skin incisions were closed and a bandage was applied.

All horses received a single dose of morphine (0.1 mg/kg btw i.v.) and antibiotics (sodium benzylpenicillin 10^7^ iu i.v.) during surgery. No medication was administered post‐operatively. Horses were box rested for 1 week after surgery, after which they were treadmill exercised for the remainder of the study period as previously described 9.

### Treatment administration

At Week 5, the intervention or control product was administered in the right MCP joint after sedation with detomidine hydrochloride (i.v., 20 μg/kg bwt). One vial of ciMSCs and one vial of EAP were thawed and drawn into one syringe, so a total volume of 2 mL was obtained. The same volume was used for the control product. Because of the nature of the intervention and control product (colour difference), this study was blinded by using separate personnel for clinical and laboratory examinations (investigators) and for administration of treatments (dispenser). One and the same investigator performed the clinical scorings throughout the study. Another investigator performed the histological scoring. For the macroscopic evaluation of the joint after euthanasia, a consensus score was derived after reaching an agreement between these two scoring investigators.

### Efficacy outcome

#### Clinical and joint assessment

**Figure 1:**
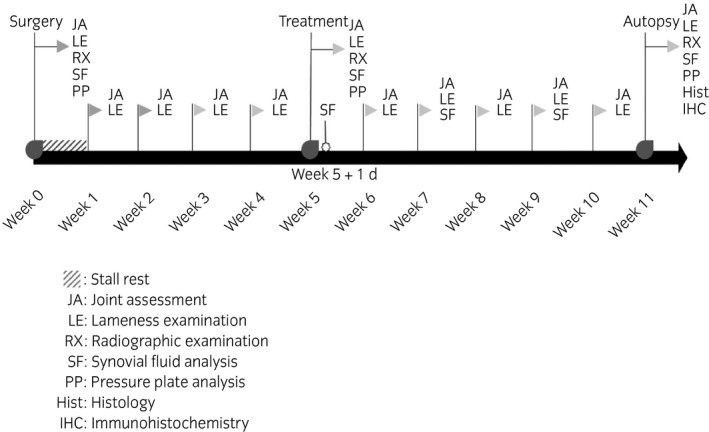
A schematic presentation of the adhered time line with an overview of the different procedures performed at each time point.

Horses underwent a daily general clinical examination and a weekly joint assessment throughout the entire study period (Fig 1). The joint assessment consisted of an evaluation of local temperature, joint effusion, pain on palpation, range of motion and measuring of the joint circumference using a measuring tape. An overview of the scoring system used for the joint assessment parameters can be found in Supplementary Item 1.

#### Lameness examination

A weekly visual and objective lameness examination was performed during the entire study period. The horses were examined on a treadmill, during lungeing on a soft surface, and in a straight line before and after distal forelimb flexion. Lameness was visually scored using the AAEP lameness scale 10. The response to flexion was scored as follows: 0 = no response to flexion, 1 = mild response to flexion, 2 = moderate response to flexion and 3 = severe response to flexion. Objective evaluation was performed using an inertial sensor‐based system (The Equinosis Q with Lameness Locator software)^d^ ^11^, ^12^. A positive vector sum represented right front limb lameness and a negative one left front limb lameness. At Weeks 0, 5 and 11, a pressure plate analysis was performed using a combination of a 2‐m pressure plate (RSscan 3D 2 m‐system)^e^ and a force plate (AMTI BP4602070RS‐2K)^f^ providing dynamic calibration of the pressure plate as previously described 13, 14. Symmetry indices were calculated and expressed as % symmetry (left/right × 100%).

#### Radiographic examination

Radiographs of both MCP joints including lateromedial, dorsopalmar and 45‐degree dorsolateral–palmaromedial and dorsomedial–palmarolateral oblique projections were taken before and the day after surgery, and at Weeks 5 and 11. Radiographic changes were recorded.

#### Synovial fluid analysis

Synovial fluid samples were collected during surgery, the day of and after treatment and at Weeks 7, 9 and 11. Cytological evaluation was performed using a haematology analyser, and viscosity was assessed using following scoring system: 0 = watery, no string, 1 = tacky, string <0.5 cm, 2 = string 0.5–4 cm and 3 = string >4 cm. The following biomarkers were determined using commercial ELISA kits according to the manufacturers’ instructions: IL‐10^g^, IL‐1 receptor antagonist protein (IRAP)^h^, PGE2^i^, MMP‐13^g^, IL‐6^j^, serum amyloid A (SAA)^g^, TNF‐α^h^, IFN‐ɣ^h^, hyaluronic acid (HA)^k^, glycosaminoglycans (GAGs)^k^^,^ l and TGF‐β3^j^.

#### Post‐mortem examination

After all examinations were performed at Week 11, all horses were subjected to euthanasia using an i.v. injection with a combination product containing embutramide (T‐61)^m^.

##### Gross examination and histology

Both MCP joints were evaluated macroscopically for cartilage and synovial health according to the guidelines of the Osteoarthritis Research Society International (OARSI) 14 as described previously 9. Cartilage was collected from both MCP joints from the area adjacent to the created fragment and at the level of the groove lesion. In addition, joint capsule and synovium were sampled. All samples were fixed in a 4% formaldehyde solution, embedded in paraffin, sectioned at 4 μm thickness and stained with haematoxylin and eosin. Cartilage repair and joint inflammation were evaluated using modified OARSI histological guidelines 15 as previously described 9. Moreover, the presence of ectopic tissue was recorded. Cartilage samples were also stained with Alcian Blue to assess GAGs content through area % calculations (see Immunohistochemistry).

##### Immunohistochemistry

Immunohistochemistry was performed on the collected tissue samples to evaluate cartilage oligomeric matrix protein (COMP), collagen type II, Ki67 and caspase 3 expression. Tissue sections were stained with rabbit polyclonal anti‐COMP (ab74524, 1:50)^g^, anti‐collagen II (ab34712, 1:50)^g^, anti‐caspase 3 (ab4051, 1:200)^g^ and mouse monoclonal anti‐Ki67 (M7240, 1:20)^n^ respectively. Immunolabelling was achieved with a high‐sensitive horseradish rabbit diaminobenzidine kit with blocking of endogenous peroxidase (Envision DAB+ kit)^o^ in an autoimmunostainer (Cytomation S/N S38‐7410‐01)^m^. Positive staining was confirmed on microscopy, and the area percentages of three randomly photographed areas (at 200× magnification) were calculated per section with the use of LAS V4.1 software^p^ ^16^.

### Data analysis

All statistical analyses were performed using SAS statistical analysis software version 9.3^q^ . The treatment groups were compared at baseline for sex using the Fisher's exact test and for age and fetlock circumference using the Wilcoxon rank‐sum test.

Data of the joint assessment, visual lameness examination, response to flexion, viscosity of synovial fluid, macroscopic and histological evaluation were compared between the treatment groups with the Cochran–Mantel–Haenszel test. Data of the joint circumference measurements, inertial sensor analysis, pressure plate analysis, biomarker analysis, synovial fluid cell counts, Alcian blue stain and immunohistochemistry were compared between the treatment groups, using the Wilcoxon signed‐rank test. A Fisher's exact test was performed to evaluate the data of the radiographic analysis (the presence or absence of radiographic changes) and the incidence of adverse events.

Since no detailed information on the different endpoints suing this treatment is available, an exact sample size could not be determined. Nevertheless, based on previous studies of our group using MSCs, a treatment success of 83% was expected, and a placebo effect of improvement of approximately 17% 7, 8. Based on a power of 80% and an alpha value of 0.05, this resulted in a sample size of 6 animals per treatment group. Moreover, this sample size has shown to generate statistical significant results in previous studies 5, 17, 18. A larger sample size was not included due to ethical considerations.

## Results

### Animals

No statistically significant differences were found between the treatment groups at day 0 for sex, age, and MCP joint circumference. For six horses (three in each treatment group), mild cartilage changes were documented during arthroscopy for the right MCP joint consisting of a superficial wear line, partial erosion, minor irregularity or thinner cartilage spots. The synovium was normal in all horses.

### Efficacy outcome

#### Lameness examination

**Figure 2:**
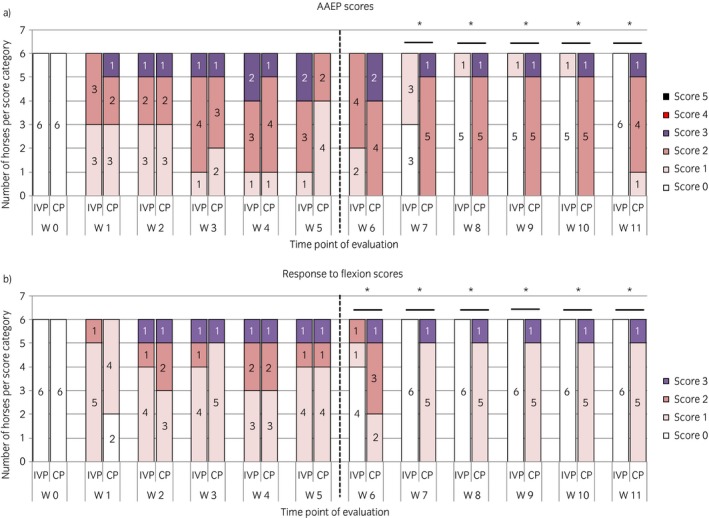
The evolution of the a) American Association of Equine Practitioners (AAEP) lameness scores and b) the response to flexion scores over the entire study period of 11 weeks for the horses treated with the placebo control product (CP) and the investigational veterinary product (IVP). The number of horses per score category is displayed per time point of evaluation. W, week. *Significant difference between the two treatment groups for that time point of evaluation with P<0.05.

There was no statistically significant difference in visual lameness scores or response to flexion between the two groups before treatment at Week 5. After treatment, AAEP scores were significantly lower with the intervention compared with the control from Week 7 onwards (P = 0.002; Fig 2a). In addition, response to flexion was also significantly lower with the intervention compared with the control from Week 6 onwards (P = 0.02 at Week 6, P = 0.001 from Weeks 7 to 11) (Fig 2b).

**Figure 3:**
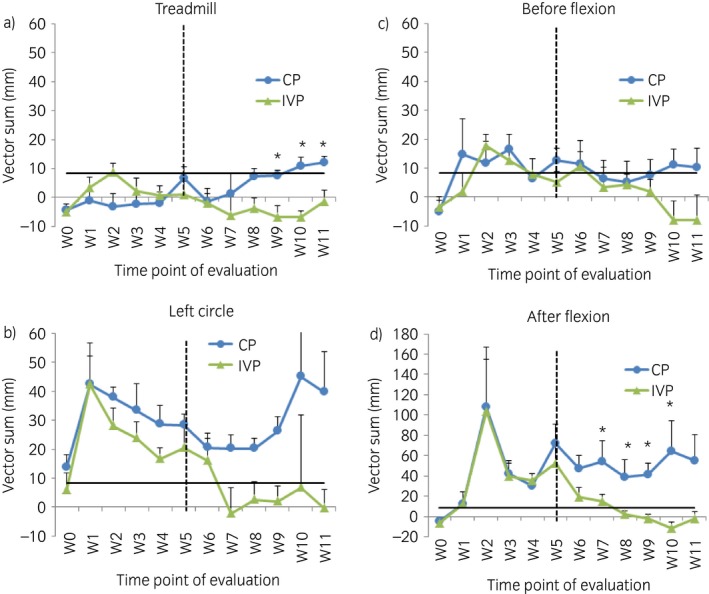
The evolution of the mean vector sums (+s.e.) measured with the inertial sensor‐based system at the different time point of evaluation on a) treadmill, b) left circle, c) straight line before flexion and d) straight line after flexion. W, week. CP, control product. IVP, investigational veterinary product. *Significant difference between the two treatment groups for that time point of evaluation with P<0.05. The black horizontal bar indicates the threshold for lameness (i.e. 8.5 mm).

There was no significant difference in average vector sums between the treatment groups before treatment administration under any of the circumstances (Fig 3). At Weeks 9, 10 and 11, the vector sums on the treadmill were significantly lower in the intervention group compared with the control group (P = 0.02) (Fig 3a). In addition, average vector sums on a straight line after flexion were also significantly lower in the intervention group from Week 7 until Week 10 (P = 0.040 for Week 7 and P = 0.017 for weeks 8, 9 and 10). At Week 11, the average vector sums were lower with the intervention than with the control (P = 0.05 for Week 11) (Fig 3d). On the left circle and on a straight line before flexion, the average vector sums were lower after treatment in the intervention group compared with the control group, especially at Week 10 and 11, but these differences were not significant (Fig 3b, c). No statistically significant differences in symmetry indices were found at any time point between the treatment groups with the pressure plate analysis.

#### Clinical assessment, joint assessment and radiographic examination

No serious adverse events or suspected adverse drug reactions occurred during the study.

Before treatment, there were five horses with no increase in local temperature and one horse with a slight increase in both the treatment groups. All horses in the intervention group reached normal local temperature at the injection site from Week 6 until the end of study. In the control group, all horses reached normal local temperature the day after treatment until the end of study.

Before treatment, there were five horses with no pain on palpation and one horse with slight pain on palpation in both the treatment groups. All horses in both the treatment groups were without pain to palpation from the day after the treatment until the end of study.

After surgery, there was a limited range of motion in one horse in the intervention group at Week 2 and in one horse of the control group at Week 3. No change in range of motion was found in any of the animals after Week 3.

**Figure 4:**
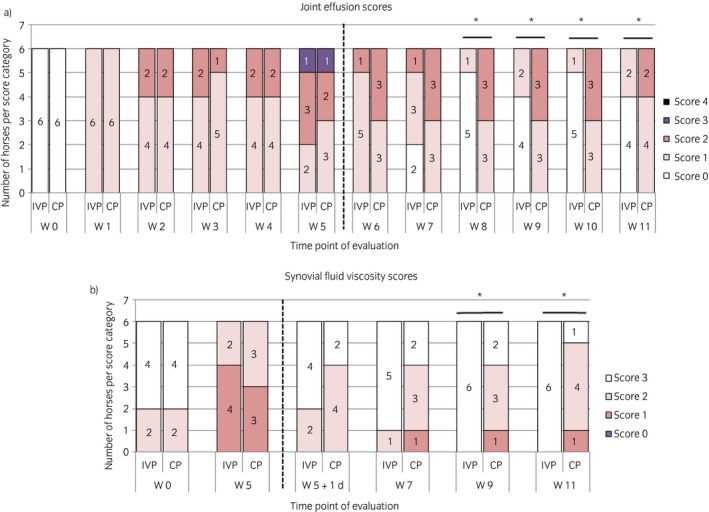
The evolution of the a) the joint effusion scores and b) the synovial fluid viscosity scores over the entire study period of 11 weeks for the placebo control product (CP)‐treated horses and the investigational veterinary product (IVP)‐treated horses. The number of horses per score category is displayed per time point of evaluation. W, week; d, day. *Significant difference between the two treatment groups for that time point of evaluation with P<0.05.

There was no significant difference in joint effusion between the groups until Week 7. From Week 8 onwards, the joint effusion scores were significantly decreased with the intervention compared with the control (P = 0.005, P = 0.01, P = 0.005 and P = 0.01 for Weeks 8, 9 10 and 11 respectively) (Fig 4a). There was no significant difference in joint circumference between the treatment groups on any study day. The number of radiographic changes was not significantly different between the two treatment groups.

#### Synovial fluid analysis

A significantly higher viscosity score was seen in the intervention group at Week 9 (P = 0.02) and at Week 11 (P = 0.006) (Fig 4b). There was, however, no significant difference between the treatment groups in total white blood cells, lymphocytes, monocytes, granulocytes and total protein at any of the time points. In addition, no significant differences were seen in IL‐10, PgE2, TGF‐β3, HA, IRAP, IL‐6, IFN‐γ, TNF‐α, SAA and MMP‐13 concentrations. However, at Week 7, the GAGs concentration in the synovial fluid was significantly lower in the intervention group compared with the control group (mean concentration (±s.d.) of 19.9 μg/mL ± 5.6 vs. 44.0 ± 28.7 μg/mL respectively P = 0.04).

#### Post‐mortem examination

##### Gross examination and histology

**Figure 5:**
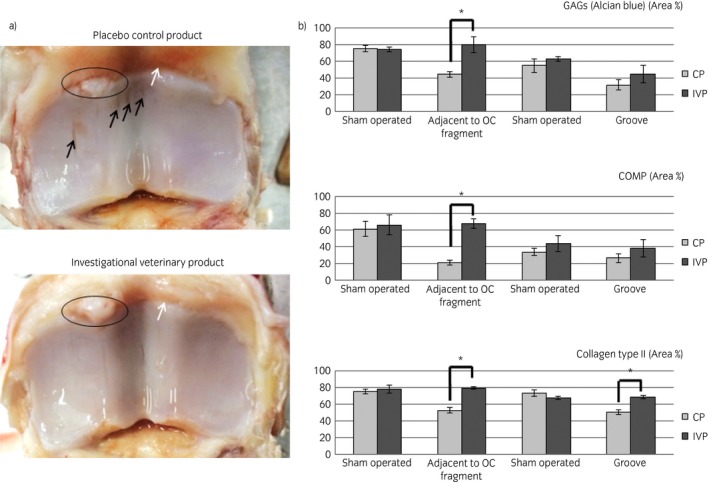
a) A representative example at gross examination of the proximal surface of the first phalanx of a horse treated with the placebo control product (CP) and a horse treated with the investigational veterinary product (IVP) (Week 11). Full thickness wear lines (indicated with the black arrows) are visible in the CP‐treated joint, but not in the IVP joint. Synovial hyperaemia (indicated with the white arrow) was also more pronounced in the CP‐treated joint compared with the IVP‐treated joint. In both joints, the dorsomedial osteochondral (OC) fragment, which was still attached to the joint capsule, was clearly visible (encircled). b) The mean area percentages (+s.e.) of glycosaminoglycans (GAGs), cartilage oligomeric matrix protein (COMP) and collagen type II as seen during histological and immunohistochemical analysis of the articular cartilage sampled adjacent to the OC fragment and at the level of the groove lesion of the CP‐ and IVP‐treated horses and of the equivalent areas in the healthy sham operated joints. *Significant difference between the treatment groups with P<0.05.

Significantly less wear lines were present in the intervention group compared with the control group on gross examination (median [range] wear line score of 0.5 1 vs. 2 2 for intervention and control respectively P = 0.02) (Fig 5a). In addition, synovitis was more prominent in the control group (median [range] of 1 1) than in the intervention group (median [range] score of 0 2), but the difference was not statistically significant (P = 0.061). Synovial hyperaemia however was significantly less pronounced in the intervention group than in the control group (median [range] score of 0 1 vs. 1 1 respectively P = 0.01) (Fig 5a). No statistically significant differences were found in erosions, extent of erosions, palmar arthrosis, covering of subchondral bone with fibrocartilage and synovial petechiation.

Histologically, there was no significant difference between the intervention and control groups in chondrocyte necrosis, cluster formation, fibrillation/fissuring, focal cell loss, cellular infiltration, vascularity, intimal hyperplasia, subintimal oedema or subintimal fibrosis. However, a significantly higher Alcian Blue uptake, an indirect measure of the amount of GAGs, was seen in the cartilage adjacent to the created OC fragment in the intervention compared with the control group (P = 0.02) (Fig 5b). No statistically significant difference was seen for the cartilage located at the groove. There was no presence of ectopic tissue in any animal.

##### Immunohistochemistry

The area % of COMP in the cartilage adjacent to the OC fragment was significantly higher in the intervention group compared with the control group (P = 0.02) (Fig 5b). The area % of COMP in the cartilage of the groove lesion was not statistically different between the two treatment groups. Collagen type II area % in the cartilage adjacent to the OC fragment and to the groove lesion was significantly higher in the intervention group than in the control group (P = 0.02) (Fig 5b). The area % of Ki67 and Caspase 3 were 0% in both the treatment groups.

## Discussion

After application of the OA model, horses clinically improved when they were treated with chondrogenic‐induced MSCs combined with equine allogeneic plasma (= intervention) compared with when they were treated with saline (= control). Indeed, a significant reduction in visual and objective lameness was seen for the intervention group together with a reduced joint effusion and improved viscosity of the synovial fluid, suggesting symptom relieve by the intervention when compared with the control treatment. Post‐mortem examination revealed a gross improvement of cartilage appearance, with a favourable local cartilage composition in the intervention group. Therefore, our results suggest an improvement of local cartilage health and metabolism 3. This improvement was also suggested by the lower concentration of GAGs in the synovial fluid of the intervention group joints. Indeed, a higher concentration of GAGs in synovial fluid indicates a release of GAGs from diseased articular cartilage into the synovial fluid, as is seen in naturally occurring OA 19, 20.

The pressure plate analysis did not reveal significant differences in the current study, probably because this analysis was performed at the end of all examinations and horses with OA generally display less lameness after warming up 21. Moreover, the pressure plate analysis only allows evaluation of a limited number of steps while the animals are trotted in a straight line over the plate. In contrast, the evaluation of multiple strides using the inertial sensor‐based system revealed a significant treatment effect under different circumstances.

Overall, the results observed in this study were more conclusive compared with those of previously reported studies on MSCs for articular cartilage repair. Wilke *et al*. 5 only found an initial improved cartilage healing after application of bone marrow‐derived MSCs combined with a fibrin vehicle in induced cartilage lesions. Frisbie *et al*. 4 evaluated adipose‐derived stromal vascular fraction and bone marrow‐derived MSCs for treatment of OA, and reported a greater improvement during various evaluations with bone marrow‐derived MSCs compared with placebo treatment and adipose‐derived stromal vascular fraction cells, but the observed effects did not reach statistical significance. McIlwraith *et al*. 6 evaluated the use of bone marrow‐derived MSCs as an addition to microfracture to augment healing of induced cartilage lesions, and reported higher quality repair tissue and a higher cumulative score during arthroscopic and gross evaluation with bone marrow‐derived MSCs. However, no clinical improvement was seen in that study. Despite the short‐term evaluation in the current study, significant effects of the intervention product were seen in the clinical, gross and histological examination. Differences between the current study and the previous studies mentioned could be related to differences in the stem cell product. In contrast to studies mentioned earlier 4, 5, 6, MSCs in the present study were chondrogenic‐induced, which has been shown to result in better cartilage adherence in explant cultures 22. In addition, a previous study from our group has shown a higher return‐to‐work‐rate when using chondrogenic‐induced MSCs combined with plasma compared with native MSCs combined with plasma 8. Moreover, the studies of Wilke *et al*. 5, Frisbie *et al*. 4 and McIlwraith *et al*. 6 used autologous stem cell preparations, while in the current study allogeneic MSCs were used. The use of an allogeneic product allowed high standardisation of the MSCs resulting in one uniform product for all horses in this study.

The intervention product we used had already been tested in an earlier target animal safety study 23. Despite the cells being allogeneic, only a transient and mild local inflammatory response was seen in that study (mild increase in lameness, heat and joint effusion), which was not significantly different from the response after a saline injection. In the present study, again no serious adverse events or suspected adverse drug reactions were seen. Moreover, the joint effusion and AAEP scores were significantly lower in the intervention group from, respectively, 3 weeks and 2 weeks after treatment administration onwards. A possible explanation of the lack of on inflammatory response to the ciMSCs used in the present study is that these cells are negative for MHC class II molecules and have a very low MHC class I expression 7. These characteristics could possibly allow the cells to be immune evasive, since it is known that both MHC I and MHC II expression on MSCs can induce an immune response in MHC‐mismatched individuals 24, 25.

In the current study, an experimental model was used to obtain standardised circumstances. However, only the short‐term effects of the intervention product were tested (6 weeks), whereas naturally occurring OA inherently presents more variability in the stage of the disease, the speed of progression and the innate healing response of the individual patient. Moreover, multiple statistical tests were performed, increasing the risk of type 1 error. Therefore, the results of this experimental study should be confirmed in a field trail on a large number of animals with naturally occurring disease.

## Conclusion

Equine allogeneic ciMSCs combined with EAP resulted in a significantly reduced lameness and joint effusion compared with a placebo treatment after a single intra‐articular injection in MCP joints with experimentally induced OA. In addition, a significantly improved synovial fluid viscosity, reduced number and/or severity of wear lines, and a decreased synovial hyperaemia was noted. Equine allogeneic ciMSCs combined with EAP also improved local cartilage composition by significantly increasing the amount of GAGs, COMP and collagen type II compared with saline‐treated control joints. Therefore, equine allogeneic ciMSCs combined with EAP could potentially be a promising treatment of OA in horses.

## Authors’ declaration of interests

J. Spaas declares competing financial interests as shareholder in Global Stem cell Technology (GST) NV. S. Broeckx, J. Spaas and L. Van Hecke are all employed by GST. S. Broeckx and J. Spaas are inventors of several pending patents owned by GST (BE2012/0656; WO2014053418A9; WO2014053420A1; PCT/EP2013/075782). The other authors declare no competing interests. The content of this manuscript contains the product Arti‐Cell^®^ Forte owned by GST.

## Ethical animal research

This study and its protocol were approved by the local ethics committee of Global Stem cell technology (approval number EC_2015_002; Permit Number: LA1700607). Collection of the donor blood was approved by the local ethics committee (approval number: EC_2012_001).

## Sources of funding

This study was supported both by a grant (number 130543) from the Global Stem cell Technology NV.

## Authorship

J. Spaas, S. Broeckx, A. Martens, F. Pille, M. Dumoulin, M. Oosterlinck, L. Duchateau, A. Bertone, F. Pille and K. Chiers conceived and designed the experiments. A. Martens performed all surgeries mentioned in the study. S. Broeckx, J. Spaas, F. Pille, L. Van Brantegem, K. Chiers, M. Dumoulin, A. Bertone, H. Hussein and M. Oosterlinck performed the data collection and implementation of the experiments. L. Duchateau performed the statistical analysis. S. Broeckx, J. Spaas, L. Van Hecke and L. Duchateau interpreted the data. S. Broeckx, L. Van Hecke and J. Spaas drafted the manuscript. All authors and approved reviewed the manuscript for submission.

## Supporting information


**Supplementary Item 1:** Clinical scoring system.

Summary in Spanish.

## Manufacturers' addresses

1 Sigma‐Aldrich, Overijse, Belgium.

2 Global Stem Cell Technology NV, Anacura Group, Evergem, Belgium.

3 B. Braun, Diegem, Belgium.

4 Equinosis, Columbia, Missouri, USA.

5 RSscan International, Paal, Belgium.

6 AMTI, Watertown, Massachusetts, USA.

7 Abcam, Cambridge, UK.

8 R&D Systems, Abingdon, Oxfordshire, UK.

9 Enzo Life Sciences, Brussels, Belgium.

10 Genorise, Glen Mills, Pennsylvania, USA.

11 Corgenix, Broomfield, Colorado, USA.

12 Astarte Biologics, Bothell, Washington, USA.

13 Intervet Belgium NV, Brussels, Belgium.

14 Agilent, Santa Clara, California, USA.

15 Dako, Leuven, Belgium.

16 Leica Microsystems, Diegem, Belgium.

17 SAS Institute Inc., Cary, North Carolina, USA.
