# Supplementary material for: The use of equine chondrogenic‐induced mesenchymal stem cells as a treatment for osteoarthritis: A randomised, double‐blinded, placebo‐controlled proof‐of‐concept study
Source: Equine Vet J. 2019 Apr 13;51(6):787–94. doi: 10.1111/evj.13089 (PMC6850029; doi:10.1111/evj.13089)
Supplement: Supplementary file 1 — Supplementary Item 1: Clinical scoring system. [file EVJ-51-787-s001.pdf]

BEVA Equine Veterinary Journal

Supplementary Item 1: An overview of the scores used during the joint and lameness assessment and an explanation of each individual score

Parameter	Score	Definition
Local temperature at injection site	0	no increased temperature sensation
	1	slightly increased temperature sensation
	2	moderate increased temperature sensation
	3	severe increased temperature sensation
Pain on palpation at local injection site	0	no pain on palpation
	1	slight pain on palpation
	2	moderate pain on palpation
	3	severe pain on palpation
Range of motion of the joint	0	normal range of motion
	1	limited (abnormal) range of motion
Joint effusion	0	none
	1	mild
	2	moderate
	3	severe
	4	extreme (peri-articular)
Response to flexion	0	no response to flexion
	1	mild response to flexion
	2	moderate response to flexion
	3	severe response to flexion
