# Supplementary material for: The use of equine chondrogenic‐induced mesenchymal stem cells as a treatment for osteoarthritis: A randomised, double‐blinded, placebo‐controlled proof‐of‐concept study
Source: Equine Vet J. 2019 Apr 13;51(6):787–94. doi: 10.1111/evj.13089 (PMC6850029; doi:10.1111/evj.13089)
Supplement: Supplementary file 2 — Summary in Spanish. [file EVJ-51-787-s002.docx]

EVJ-GA-18-179.R3

El uso de células madres condrogénicas mesenquimatosas inducidas como tratamiento de la osteoartritis: un estudio de prueba de concepto aleatorio, doble ciego, controlado con placebo.

S. Y. Broeckx^1,2¶^, A. M. Martens^2,¶^, A. L. Bertone^3^, L. Van Brantegem^4^, L. Duchateau^5^, L. Van Hecke^1^, M. Dumoulin^2^, M. Oosterlinck^2^, K. Chiers^4^, H. Hussein^3^, F. Pille^2,*^ and J. H. Spaas^1*^

**Palabras clave:** caballo; alogénico; articulación metacarpo falángica; modelo; sangre periférica

**Resumen**

**Historial:** Existe una necesidad de mejorar terapias para la osteoartritis en caballos.

**Objetivos:** Para asesorar la eficacia de células madres equinas alogénicas condrogénicas inducidas, combinadas con plasma como una nueva terapia para la osteoartritis en caballos.

**Diseño del estudio:** Experimento aleatorio, doble ciego, controlado con placebo.

**Métodos:** En 12 caballos sanos, fue inducida osteoartritis en la articulación metacarpo falángica usando un modelo de fragmento de surco osteocondral. A las cinco semanas después de la cirugía, a los caballos se les asigno aleatoriamente una inyección de células madres condrogénicas inducidas + plasma equino alogénico (=intervención) o con 0.9% solución salina (=control). Desde la cirugía hasta el final del estudio, los caballos fueron sometidos a evaluaciones semanales de las articulaciones y de cojera. Se tomaron muestras de líquido sinovial para citología y análisis de biomarcadores antes de la cirugía y a la semana 5, semana 5+1 día, semana 7, semana 9 y semana 11. A la semana 11, los caballos fueron eutanasiados y las articulaciones metacarpo falángicas fueron evaluadas macroscópicamente e histológicamente.

**Resultados:** Ningún evento adverso o sospecha de reacción adversa a droga ocurrieron durante el estudio. Una mejora significativa de la cojera visual y objetiva se vio con la intervención en comparación con el control. El líquido sinovial mostró tener una mayor viscosidad y una concentración significativamente menor de glucosaminoglicanos en el grupo con intervención. Los otros biomarcadores o parámetros citológicos no fueron significativamente diferentes entre los grupos de tratamientos. Significativamente menos líneas de desgaste y de hiperemia sinovial estaban presentes en el grupo con intervención. La cantidad de proteína matriz de cartílago oligométrico, colágeno tipo II y glucosaminoglicanos fueron significativamente mayores en el cartílago articular del grupo con intervención.

**Limitaciones principales:** Este estudio evaluó el efecto a corto plazo de la intervención en un número limitado de caballos, usando un modelo de osteoartritis. Este estudio también incluyo pruebas estadísticas múltiples, aumentado el riego de error tipo 1.

**Conclusiones:** Células madres mesenquimatosas alogénicas equinas inducidas combinadas con plasma equino alogénico podría ser un tratamiento prometedor para osteoartritis en el caballo.
